# NIR Photoregulated Theranostic System Based on Hexagonal-Phase Upconverting Nanoparticles for Tumor-Targeted Photodynamic Therapy and Fluorescence Imaging

**DOI:** 10.3390/nano10122332

**Published:** 2020-11-25

**Authors:** Linlin Zhao, Jongseon Choi, Yan Lu, So Yeon Kim

**Affiliations:** 1 Tianjin Key Laboratory for Photoelectric Materials and Devices, School of Materials Science & Engineering, Tianjin University of Technology, Tianjin 300384, China; luxingzhao@hotmail.com; 2 Graduate School of Energy Science and Technology, Chungnam National University, Daejeon 34134, Korea; powercjs@naver.com; 3 Department of Chemical Engineering Education, College of Education, Chungnam National University, Daejeon 34134, Korea

**Keywords:** upconversion nanoparticle, photoluminescence, photodynamic therapy, fluorescence imaging, theranostic

## Abstract

Although photodynamic therapy (PDT) is an effective, minimally invasive therapeutic modality with advantages in highly localized and specific tumor treatments, large and deep-seated cancers within the body cannot be successfully treated due to low transparency to visible light. To improve the therapeutic efficiency of tumor treatment in deep tissue and reduce the side effects in normal tissue, this study developed a near-infrared (NIR)-triggered upconversion nanoparticle (UCNP)-based photosensitizer (PS) carrier as a new theranostics system. The NaYF_4_:Yb/Er UCNPs were synthesized by a hydrothermal method, producing nanoparticles of a uniformly small size (≈20 nm) and crystalline morphology of the hexagonal phase. These UCNPs were modified with folic acid-conjugated biocompatible block copolymers through a bidentate dihydrolipoic acid linker. The polymer modified hexagonal phase UCNPs (FA-PEAH-UCNPs) showed an improved dispersibility in the aqueous solution and strong NIR-to-vis upconversion fluorescence. The hydrophobic PS, pheophorbide a (Pha), was then conjugated to the stable vectors. Moreover, these UCNP-based Pha carriers containing tumor targeting folic acid ligands exhibited the significantly enhanced cellular uptake efficiency as well as PDT treatment efficiency. These results suggested that this system could extend the excitation wavelength of PDT to the NIR region and effectively improve therapeutic efficiency of PSs.

## 1. Introduction

Photodynamic therapy (PDT) has been recognized as a promising treatment method for a variety of cancers due to its minimal normal tissue toxicity, little invasiveness, highly localized and specific tumor treatments, fewer adverse effects as compared with chemotherapy and radiation therapy [1,2,3,4]. PDT is a powerful noninvasive therapeutic technique for a range of diseases including cancers, based on the photochemical reactions mediated by the interaction of photosensitizers (PSs) with a particular type of light and molecular oxygen. When PSs are exposed to a specific wavelength of light, the PS molecules are activated and transfer energy to oxygen in the surrounding tissue, generating active forms of oxygen as singlet oxygen (^1^O_2_). The highly reactive oxygen species (ROS) can react with biological molecules, resulting in an irreversible oxidative tissue damage and cell death [5,6,7,8].

However, most clinically approved current PSs in PDT are hydrophobic and hence have limited solubility in aqueous solution. These hydrophobic PSs can easily aggregate under physiological conditions, which can dramatically decrease the ^1^O_2_ quantum yield and eventually affect the therapeutic efficiency of PDT [9,10]. To address these issues, various nanocarriers such as micelles [11,12], liposomes [13], dendrimers [14], gold nanoparticles [15], mesoporous materials [16] and carbon nanotubes [17,18,19] have been investigated as for the delivery of PSs in cancer therapy.

In addition, another main challenge for PDT is efficient treatment of cancers at a deep tissue level. However, the PSs used in conventional PDT are usually excited by high energy visible or UV light, which limits the penetration depth in biological tissues. PDT has been generally applicable to tumors on or just under the skin or on the lining of internal organs but does not produce effective therapeutic effects when treating large and deep-seated tumors [20,21].

Near-infrared (NIR) light is considered to be a transparency window of the biological tissues due to the minimal light absorption and scattering. Compared with the UV or visible light, NIR shows lower photodamage effects, greater tissue penetration depth, and higher signal-to-noise ratio [22,23]. However, the current PSs for clinical usage, which can be efficiently activated by NIR light, remain rare.

Upconversion is an optical process that involves the conversion of lower-energy photons into higher-energy photons [24,25,26]. Especially, lanthanide ion-doped upconversion nanoparticles (UCNPs) show unique luminescent properties, including the ability to convert NIR excitation radiation into high-energy visible and ultraviolet emission through a process known as photon upconversion. This process can further activate the PSs attached to UCNPs to produce ROS [27,28,29]. The use of UCNPs in PDT will enable full utilization of current and commercial PSs upon NIR irradiation [30,31,32]. In particular, UCNPs with a hexagonal phase have been proven to be excellent NIR-to-visible nanotransducers, which could provide the high photon upconversion efficiency [24,25,26]. 

**Figure 1:**
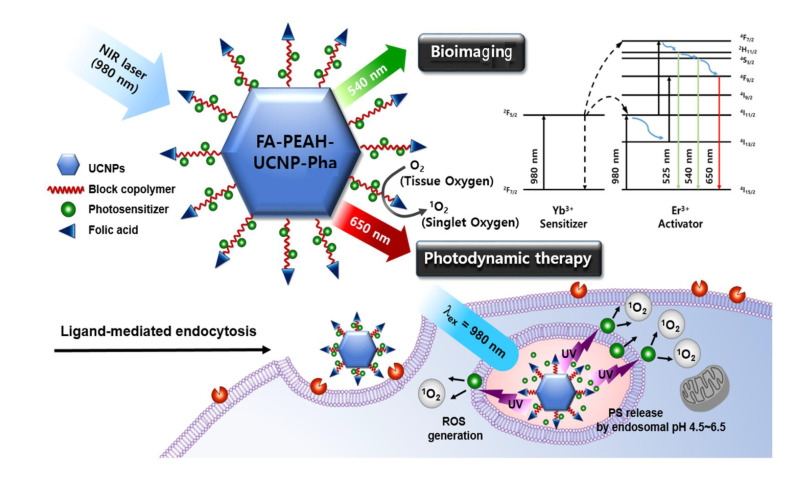
Schematic illustration demonstrating the photodynamic therapy and upconversion mechanism of pH-responsive polymer modified (FA-PEAH)-upconversion nanoparticles (UCNPs)- pheophorbide a (Pha).

Recently, the UCNP-based PS delivery system for PDT has widely attracted interest from scientists, as it shows potential to overcome the above-mentioned drawbacks of current PDT [33,34,35,36]. However, there are still technical difficulties in the practical application of UCNP-based PS carriers, such as the inadequate quantum yield of UCNPs at an excitation density that is safe to human skin [37]. In addition, the strategy of a UCNP-based theranostics system with a tumor-targeting ligand for selective PS delivery has been reported in only a few publications [38]. Therefore, we aimed to develop a NIR-regulated theranostic system based on hexagonal-phase UCNPs for tumor-targeted PDT and fluorescence imaging as shown in Figure 1.

In this study, we optimized the hydrothermal synthesis procedure to produce NaYF_4_:Yb/Er UCNPs with uniform size, hexagonal phase, and strong fluorescent intensity. In order to increase the aqueous solubility of UCNPs and introduce functional moieties into the surface of UCNPs for subsequent biological functionalization, folic acid-polyethylene glycol-poly(aspartic acid-hydrazone)-dihydrolipoic acid (FA-PEAH) polymer chains were conjugated. Then, pheophorbide a (Pha), was conjugated to the side chain of FA-PEAH copolymer via an acid-labile hydrazone linker, which remained intact and stable at physiological pH, but decomposed at the lower pH of the endosomal/lysosomal compartments. The size, size distribution, elemental composition, crystalline morphology, and luminescence properties of UCNPs were determined. To assess the potential of FA-PEAH-UCNPs-Pha as a NIR-triggered theranostic system, in vitro cellular localization and phototoxicity effects of UCNP-based nanocarriers were also investigated.

## 2. Materials and Methods

### 2.1. Materials

Yttrium(III) chloride hexahydrate (YCl_3_·6H_2_O), erbium(III) chloride hexahydrate (ErCl_3_·6H2O), ytterbium(III) chloride hexahydrate (YbCl_3_·6H_2_O), ammonium fluoride (NH_4_F), 4-(dimethylamino) pyridine (DMAP), and triphosgene oleic acid were purchased from Aldrich Chemical Co. (Milwaukee, WI, USA). Sodium borohydride, *N,N’*-dicyclohexylarbodiimide (DCC), and *N*-hydroxysuccinimide (NHS) were obtained from Fluka (Buchs, Switzerland). 4-Hydroxy-2-butanone was purchased from TCI (Tokyo, Japan). α-Lipoic acid (LA), *N*-(3-dimethylaminopropyl)-*N′*-ethylcarbodiimide hydrochloride (EDC), folic acid (FA), β-benzyl-L-aspartate (BLA), triethylamine (TEA), hydrazine monohydrate, PEG-bis(amine) (molecular weight: 3.350 kDa), sodium bicarbonate were purchased from Sigma Chemical Co. (St. Louis, MO, USA). Tetrahydrofuran (THF), n-hexane, benzene, *N,N*-dimethylformamide (DMF), methanol, chloroform, diethyl ether, dimethyl sulfoxide (DMSO), 1, 4-dioxane, acetic acid, and dichloromethane (DCM) were obtained from Samchun Pure Chemical Co., Ltd. (Gyeonggi-do, Korea). Pheophorbide a (Pha) was obtained from Frontier Scientific, Inc. (Logan, UT, USA).

### 2.2. Synthesis of Tumor-Targeted Ligand and Photosensitizer-Conjugated UCNPs

#### 2.2.1. Synthesis of Hexagonal Phase NaYF4:Yb/Er UCNPs

YbCl_3_·6H_2_O (0.18 mmol), ErCl_3_·6H_2_O (0.02 mmol), and YCl_3_·6H_2_O (0.8 mmol) were mixed with oleic acid (25 mL) in a 250 mL flask. To form a homogeneous solution, the solution was heated to 160 °C and then cooled to room temperature. A 10 mL methanol solution containing NH_4_F (4.0 mmol) and NaOH (2.5 mmol) was slowly added into the flask and stirred for 30 min. The solution was heated to 100 °C to evaporate the methanol, and then heated to 300 °C and maintained for 1 h under an N_2_ atmosphere. After the solution was cooled naturally to room temperature, the resulting materials were precipitated from the solution using ethanol and then washed three times with a water/ethanol mixture (*v*/*v* = 1:1) [39].

#### 2.2.2. Surface Modification of UCNPs

FA-conjugated block copolymer (FA-PEAH) composed of PEG, FA, poly(aspartate), and a dihydrolipoic acid end group, for surface modification of UCNPs was prepared as we reported previously [40]. The surface modification of UCNPs was performed by a ligand exchange method using the synthesized FA-PEAH block copolymers. The UCNPs (80 mg) were dispersed in 10 mL aqueous solution. The dispersion was stirred for 2 h while maintaining the pH at 4 by adding 0.1 M HCl solution to remove the oleate ligands. To remove the oleic acid by extraction with hexane, the solution was mixed with hexane, and repeated three times. The combined hexane layers were re-extracted with water. In addition, the water layers were combined and re-extracted with hexane. After precipitation with cold acetone, the ligand-free UCNPs in the water dispersible fraction were collected by centrifugation. The product was re-dispersed in acetone and then the particles were collected by centrifugation. Finally, the particles were dispersed in water (20 mL) for future use. FA-PEAH (160 mg) was dissolved in 10 mL water, and the solution was added into ligand-free UCNP aqueous solution. The mixture was stirred at room temperature for 24 h, and then purified by dialysis against deionized water for 6 h. The resulting product was freeze-dried for further study [41,42].

#### 2.2.3. Preparation of Pha-Conjugated UCNP Nanocarriers (FA-PEAH-UCNPs-Pha)

To introduce the ketone groups to Pha molecules, Pha (0.176 mol) was dissolved in 30 mL methanol. 4-Hydroxy-2-butanone (1.39 mmol), EDC (0.53 mmol), and DMAP (0.53 mmol) were added to the Pha solution. The mixture was stirred at 400 rpm for 24 h in a dark place at room temperature. Next, the solvent from the resulting mixture was removed in a vacuum oven, and the residue was washed with deionized water. The ketone group-modified Pha (Pha-HB) product was collected by centrifugation, and then freeze-dried.

Next, FA-PEAH-modified UCNPs (120 mg) were dissolved in DMSO (10 mL). The modified Pha (40 mg) dissolved in 4 mL DCM was added into the FA-PEAH-UCNPs solution. Subsequently, four drops of acetic acid were added into the mixture. The reaction mixture was stirred for 24 h without light interference under room temperature. After the DCM was removed under vacuum, DMSO was added into the residue (3 mg/mL). The solution was dialyzed against NaHCO_3_ (pH 8.0) solution for 1 day, and then against deionized water for another 12 h. The resulting products were collected by centrifugation, and then the products were freeze-dried. In addition, an FA-unconjugated UCNP carrier sample (CH_3_-PEAH-UCNPs-Pha) was prepared using a similar method as a control group.

### 2.3. Characterization

The modification of copolymers was determined by 600 MHz ^1^H NMR (AVANCE III 600, Bruker, Rheinstetten, Germany) using D_2_O, and DMSO-d_6_ as the solvent. Size distributions and sizes of UCNPs were measured by dynamic light scattering (ELS-Z, OTSUKA, Hirakata, Japan) at 25 °C using a He-Ne laser (633 nm) as a light source. The surface modification of UCNPs was measured by UV-visible spectrophotometry (UVmini-1240, Shimadzu, Japan), and energy dispersive spectroscopy (EDS) (Tecnai G^2^ F30 TEM system). The morphologies of the UCNPs were observed by field-emission transmission electron microscopy (FE-TEM) (Tecnai G^2^ F30, FEI, Amsterdam, the Netherlands). The crystalline morphology of nanoparticles was also investigated by selected area electron diffraction (SAED) and X-ray Diffraction (XRD) (D8 DISCOVER, Bruker, Rheinstetten, Germany).

### 2.4. Detection of Singlet Oxygen Generation

The generation of singlet oxygen from FA-PEAH-UCNPs-Pha was determined under 980 nm laser irradiation by monitoring the photobleaching of 1,3-diphenylisobenzofuran (DPBF), that is bleached by singlet oxygen. Experiments were carried out in a quartz cuvette containing 3 mL of FA-PEAH-UCNPs-Pha solution (12 μM) in acetonitrile, and 150 μL of DPBF solution (2 mM). The optical density at 411 nm (λ_max_ of DPBF) was monitored every 2 min using UV–visible spectrometer as a function of time.

### 2.5. Cellular Uptake of FA-PEAH-UCNPs-Pha

The MCF7 breast cancer cell is a FA receptor-overexpressing cell line [43,44], used here for cellular uptake and phototoxicity tests. MCF7 cells were seeded onto 6-well culture plates (1 × 10^5^ cells/well) and cultured in RPMI 1640 supplemented with 1% penicillin-streptomycin and 10% FBS at 37 °C under a humidified atmosphere with 5% CO_2_. After incubation for 24 h, the medium was replaced with 1.5 mL of fresh medium containing FA-PEAH-UCNPs-Pha (10 μg/mL Pha equiv.) and free Pha (10 μg/mL), and then incubated for 4 h. The cell nucleus and F-actin were stained by using 4′,6-Diamidine-2-phenylindol (DAPI) and Alexa Fluor 488 phalloidin. All experiments were performed without light interference under room temperature to prevent photodegradation of the probes. The stained cells were observed using a confocal laser scanning microscope (LSM800, Carl-Zeiss, Jena, Germany).

### 2.6. In Vitro Phototoxicity Assay of FA-PEAH-UCNPs-Pha

MCF7 cells were seeded onto 96-well culture plates and cultured in RPMI 1640. The cell density was about 1 × 10^4^ cells per well. After 24 h, the medium was replaced with RPMI 1640 medium containing free Pha and FA/CH_3_-PEAH-UCNPs-Pha under a series of concentrations (0, 5, 10, 20, and 30 μg/mL, Pha equiv.) with 980 nm laser treatment at 0.1 mW/cm^2^ for 5 min. After incubation for 24 h, the cell viability was evaluated using a cell viability assay kit (CCK-8, DoGenBio, Korea). Untreated cells served as 100% viable cells. To determine the effect of laser exposure time on the phototoxicity, we also evaluated the in vitro phototoxicity of free Pha, FA-PEAH-UCNPs-Pha, and CH_3_-PEAH-UCNPs-Pha samples after laser (980 nm) radiation for 0, 0.5, 1, and 5 min at 0.2 mW/cm^2^, the concentration of Pha was selected at 10 μg/mL (Pha equiv.). Dark-toxicity of the FA-PEAH-UCNPs-Pha was also investigated by incubating for 4 h under 10 μg/mL (Pha equiv.) without laser irradiation.

## 3. Results and Discussion

### 3.1. Synthesis and Characterization of the UCNP-Based Nanocarrier

**Figure 2:**
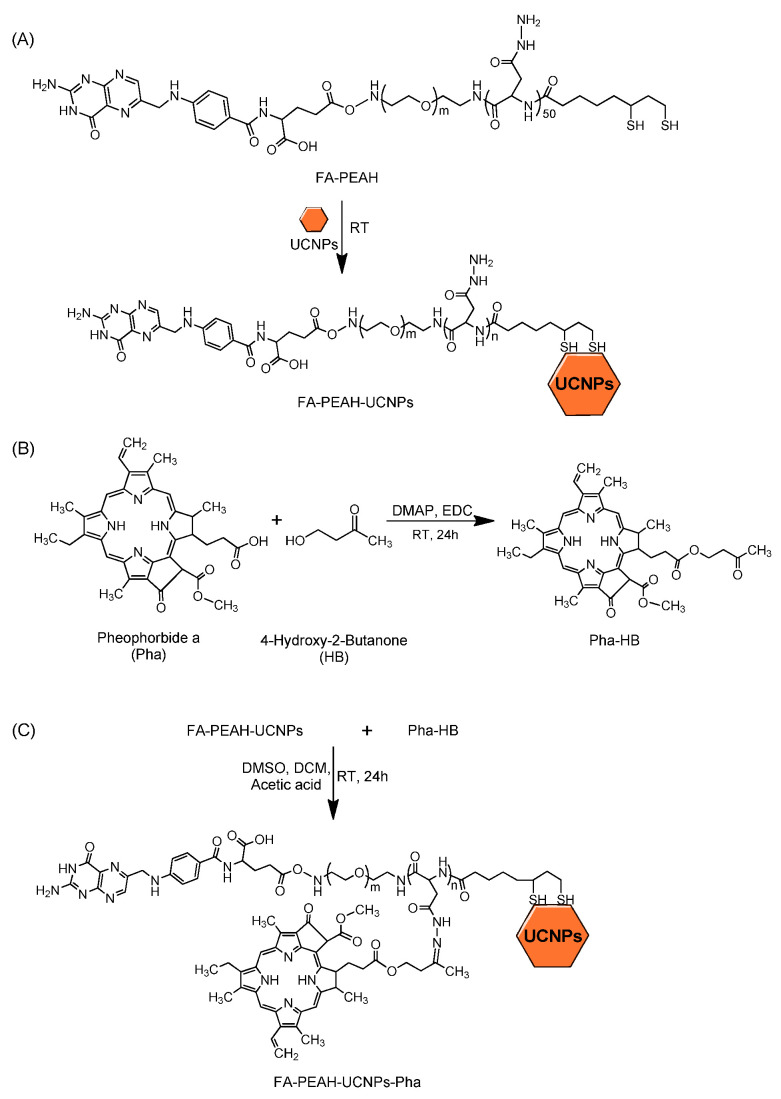
Synthesis of (**A**) polymer modified UCNPs (FA-PEAH-UCNPs), (**B**) ketonized Pha (Pha-HB), and (**C**) FA-PEAH-UCNPs-Pha.

The synthetic scheme of the FA-PEAH-UCNPs-Pha nanocarrier is illustrated in Figure 2. Characterization of the FA-PEAH block copolymer used for UCNPs modification was described in detail in our previous report [40].

**Figure 3:**
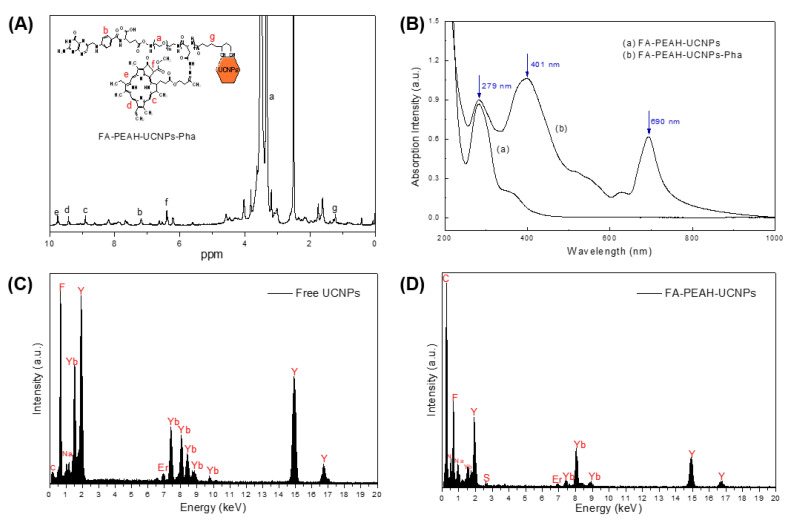
(**A**) ^1^H NMR spectra of FA-PEAH-UCNPs-Pha; (**B**) UV-visible absorption spectra of (a) FA-PEAH-UCNPs and (b) FA-PEAH-UCNPs-Pha; and (**C**) EDS spectra of free UCNPs; (**D**) EDS spectra of FA-PEAH-UCNPs).

As shown in Figure 3A, the synthesis of Pha-conjugated UCNP nanocarrier (FA-PEAH-UCNPs-Pha) was confirmed by the presence of the characteristic peaks of Pha at 8.9 ppm, 9.4 ppm, and 9.8 ppm, as well as characteristic peaks of FA at 7.2 ppm, PEG at 3.5 ppm, P(Asp) at 8.2 ppm, and DHLA at 1.2 ppm. In addition, the conjugation content of Pha to FA-PEAH-UCNPs was determined by ^1^H NMR using the relative intensity ratio of the methylene protons of the PEG chain (-OCH_2_CH_2_-, 3.5 ppm) to the methylene protons of Pha (8.9 ppm, 9.4 ppm, and 9.8 ppm). The conjugation content of photosensitizer Pha in FA-PEAH-UCNPs-Pha carrier was determined as about 14.7%.

Furthermore, the formation and surface modification of lanthanide-doped NaYF4:Yb/Er UCNPs were characterized by UV-visible spectroscopy and EDS measurements. Figure 3B showed the UV-visible absorption spectra of FA-PEAH-UCNPs and FA-PEAH-UCNPs-Pha. As shown in Figure 3B, the peak at about 279 nm was assigned to FA, while the peaks at about 401 nm, and 690 nm were attributed to Pha. This observation indicated that the Pha was introduced successfully to FA-PEAH polymer chain.

EDS was also employed to evaluate the elemental composition of UCNPs before and after surface modification. As shown in Figure 3C, the characteristic peaks of F, Na, Yb, Y, Er, and C were observed in the free UCNPs sample. After FA-PEAH modification on the surface of UCNPs by a ligand cap exchange method, new S and N characteristic peaks belonging to the FA-PEAH copolymer appeared, and the relative intensity of the C peak increased significantly (Figure 3D). These results indicated that the NaYF_4_:Yb/Er UCNPs were formed and the FA-PEAH layer was successfully immobilized onto the surface of UCNPs. After surface modification, the solubility of UCNPs improved significantly at the macroscopic level.

### 3.2. Morphology of UCNP-Based Nanocarriers

Size distribution, size, morphology and crystalline morphology of free UCNPs, FA-PEAH-UCNPs, and FA-PEAH-UCNPs-Pha were determined by DLS, FE-TEM, SAED and XRD.

**Figure 4:**
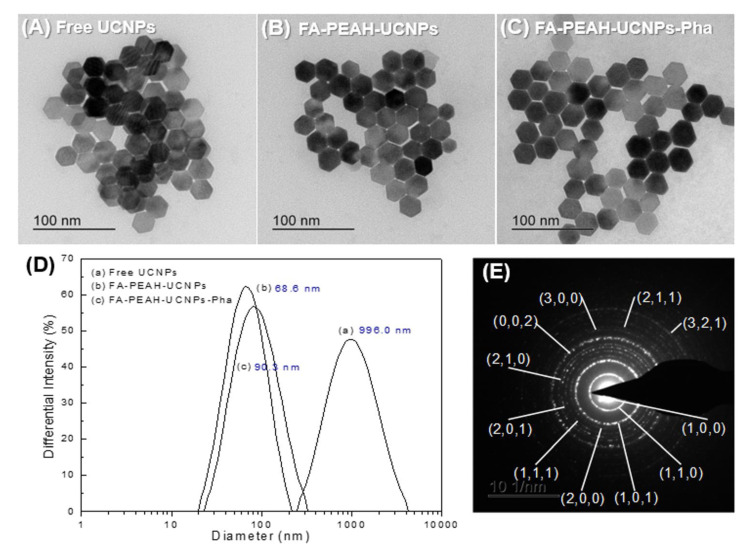
TEM images of (**A**) free UCNPs, (**B**) FA-PEAH-UCNPs, and (**C**) FA-PEAH-UCNPs-Pha; (**D**) Typical size distributions of UCNPs, (a) Free UCNPs, (b) FA-PEAH-UCNPs (b), FA-PEAH-UCNPs-Pha; (**E**) selected area electron diffraction (SAED) pattern of NaYF_4_:Yb/Er UCNPs synthesized by a hydrothermal method.

The morphologies of UCNPs, FA-PEAH-UCNPs and FA-PEAH-UCNPs-Pha were observed by FE-TEM. As shown in Figure 4A–C, these NaYF_4_:Yb/Er nanocrystals were uniform submicron in size and monodisperse size distribution. The particle size was about 20 nm in dimeter with a hexagonal plate-like shape. The sizes of UCNPs were almost the same before and after modification in TEM images as shown in Figure 4A–C, while the sizes before and after modification were quite different in the DLS data (Figure 4D). Since TEM measurement is sensitive only to the electron dense metal particles, the size of all samples in the TEM images were almost the same and the polymers used for surface modification were not clearly observed. However, DLS measurement is sensitive to the hydrodynamic diameter of the whole nanocomposite. Thus, the DLS results of surface-modified UCNPs, FA-PEAH-UCNPs and FA-PEAH-UCNPs-Pha samples, exhibit a larger size than the TEM results.

Typical average particle size distributions measured by DLS for free UCNPs, FA-PEAH-UCNPs, and FA-PEAH-UCNPs-Pha are shown in Figure 4D as 996.0, 68.6, and 90.3 nm, respectively. Since free UCNPs were quite hydrophobic due to the hydrophobic oleate capping ligand before surface modification, the macroscopic aggregations were observed. Thus, DLS data of free UCNPs exhibited a much larger size compared with the surfaced-modified UCNP samples (FA-PEAH-UCNPs and FA-PEAH-UCNPs-Pha). However, after surface modification of the hydrophilic FA-PEAH polymer instead of the hydrophobic oleate ligand, the dispersity and solubility in aqueous solution significantly improved. As shown in Figure 4D, the size of the nanoparticles significantly decreased (about 68.6 nm for FA-PEAH-UCNPs and 90.3 nm for FA-PEAH-UCNPs-Pha), and the size distribution maintained a narrow monodisperse unimodal pattern.

**Figure 5:**
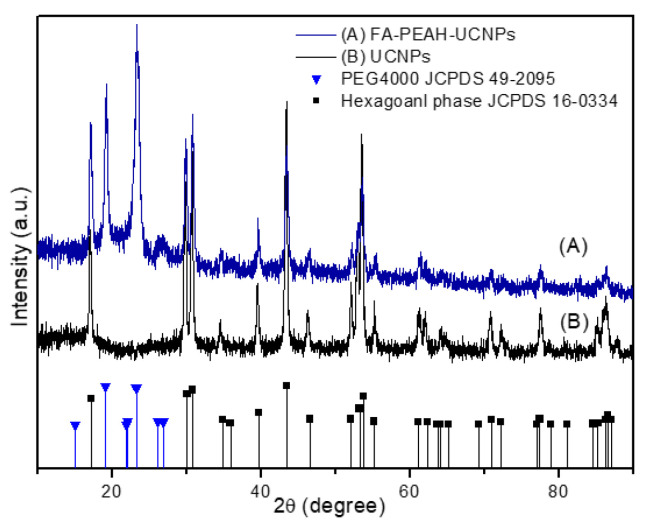
XRD patterns of (A) hexagonal-phase NaYF_4_:Yb/Er UCNPs, and (B) copolymer-modified hexagonal-phase NaYF_4_:Yb/Er UCNPs (FA-PEAH-UCNPs), bottom line patterns are the hexagonal-phase (JCPDS 16-0334) and PEG 4000 (JCPDS 49-2095).

In order to evaluate the deep-penetration PDT application, hexagonal-phase UCNPs are the best choice, because hexagonal-phase NaYF_4_:Yb/Er UCNPs usually produce a bright green emission around 550 nm along with a weak dark red emission around 660 nm under 980 nm NIR irradiation [45]. It has been also reported that NaYF_4_ crystals with hexagonal-phase (β-NaYF4) are the most efficient host materials for upconverting lanthanide ions due to the low phonon energy of the crystal lattice. The crystalline morphology of the synthesized NaYF_4_:Yb/Er UCNPs was investigated by the SAED pattern, as shown in Figure 4E. The SAED pattern of the UCNPs was shown as spotty polycrystalline diffraction rings, which can be indexed to the (100), (110), (101), (110), (200), (111), (201), (210), (002), (300), (211), and (321) planes of hexagonal NaYF_4_ lattice. We also employed XRD to further confirm the crystalline morphology of NaYF_4_:Yb/Er UCNPs. In Figure 5, the peak positions and intensities of the free UCNPs agree well with the standard pattern of hexagonal phase NaYF_4_ crystal (Figure 5; ■, JCPDS 16-0334). These results indicated that the synthesized NaYF_4_:Yb/Er UCNPs have the hexagonal β-phase. Additionally, in Figure 5, the peak positions between 15 and 30 degrees were well matched with the standard pattern of PEG (▼, JCPDS 49-2095). It also could be evidence that the FA-PEAH layer was successfully immobilized onto the surface of UCNPs.

### 3.3. Luminescence Properties of UCNPs

**Figure 6:**
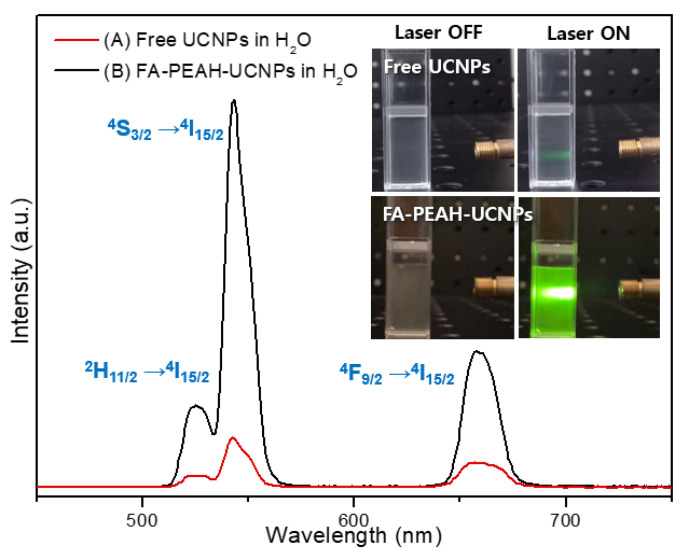
Fluorescence emission spectra of (A) free UCNPs (B) copolymer-modified UCNPs (FA-PEAH-UCNPs) in water under 980 nm laser excitation; inset shows the green fluorescence emission from free UCNPs and FA-PEAH-UCNPs upon 980 nm laser excitation.

The upconversion fluorescence spectra of free UCNPs and surface modified UCNPs (FA-PEAH-UCNPs) in aqueous solution excited with a 980 nm laser at room temperature are shown in Figure 6. As shown in Figure 6, the luminescence efficiency of FA-PEAH-UCNPs increased significantly compared to free UCNPs after surface modification. The free UCNPs stabilized by oleic acid had very weak luminescence intensity because they were rarely dispersed in aqueous solution, whereas the dispersion properties of FA-PEAH-UCNPs increased and the luminescence intensity increased significantly after modifying the water-soluble FA-PEAH. 

The FA-PEAH-UCNPs samples show three distinct Er^3+^ emission bands. The sharp green emissions at 510~530 nm and at 530~570 nm were assigned to the ^2^H_11/2_ → ^4^I_15/2_ and ^4^S_3/2_ → ^4^I_15/2_ transitions, respectively. In addition, a red emission was observed at 645~680 nm corresponding to the ^4^F_9/2_ → ^4^I_15/2_ transition. The inset in Figure 6 exhibits photographs of free UCNPs and FA-PEAH-UCNPs in aqueous solution under 980 nm laser irradiation. The free UCNPs and FA-PEAH-UCNPs sample show a yellowish green color upon excitation by a 980 nm laser. The red emission band of UCNPs between 645 and 680 nm exhibits a good match for the main absorption band of Pha between 645 and 735 nm. This result indicates that the photosensitizer Pha molecules could be activated by luminescence intensity of FA-PEAH-UCNPs upon 980 nm laser irradiation.

### 3.4. Detection of Singlet Oxygen Generation

**Figure 7:**
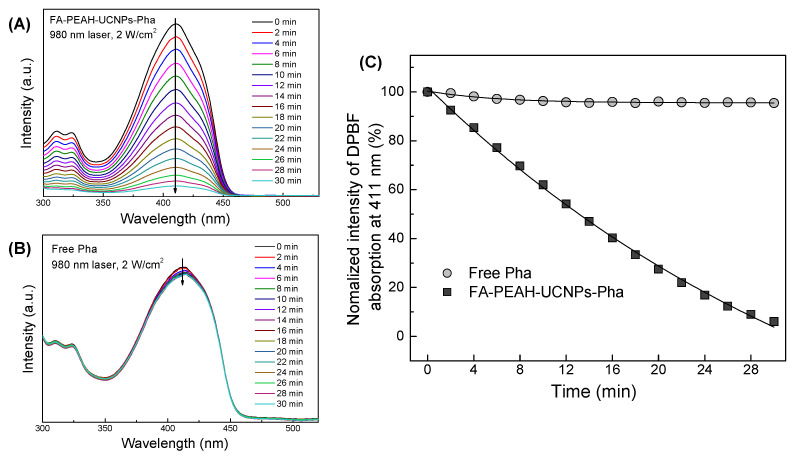
(**A**) The decrease in absorbance intensity of DPBF at 411 nm at different irradiation time of FA-PEAH-UCNPs-Pha, (**B**) the decrease in absorbance intensity of DPBF at 411 nm at different irradiation time of free Pha, and (**C**) singlet oxygen generation from free Pha and FA-PEAH-UCNPs-Pha detected by using DPBF as a function of 980 nm laser irradiation time.

The generation of singlet oxygen from FA-PEAH-UCNPs-Pha was determined under 980 nm laser irradiation by monitoring the photobleaching of 1,3-diphenylisobenzofuran (DPBF), that is bleached by singlet oxygen. The decrease in absorbance peak intensity of DPBF at 411 nm was monitored every 2 min using UV–visible spectrometer as a function of time (Figure 7A,B). FA-PEAH-UCNPs-Pha sample showed a sharp decline in optical density at 411 nm (λ_max_ of DPBA), indicating rapid generation of singlet oxygen as a function of time of 980 nm laser (2 W/cm^2^) exposure. On the other hand, the DPBF absorbance of free Pha as a function of time has not changed significantly since free Pha was not activated by 980 nm NIR laser. In the case of FA-PEAH-UCNPs-Pha, the absorbance of DPBF decreased by about 6% within 30 min. These results indicate that the luminescence intensity of FA-PEAH-UCNPs-Pha by NIR 980 nm laser irradiation can activate the Pha molecules and produces singlet oxygen leading to cytotoxicity.

### 3.5. Cellular Localization of UCNP-Based Nanocarrier in Tumor Cells

**Figure 8:**
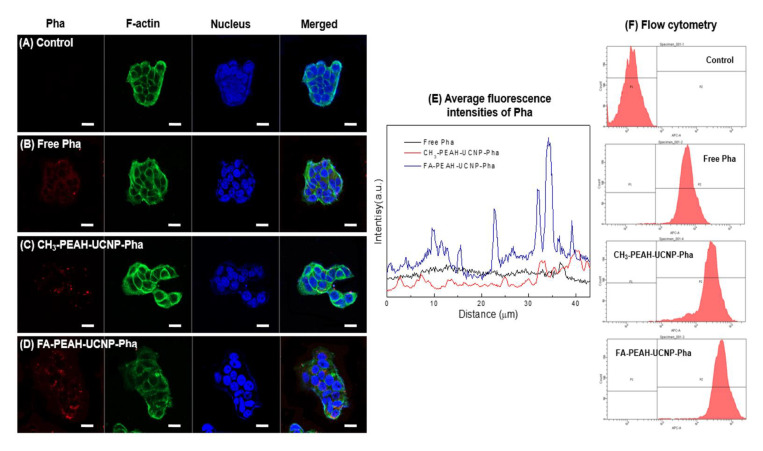
Confocal images of free Pha and FA/CH_3_-PEAH-UCNPs-Pha against MCF7 cells (DAPI (blue color), F-actin (green color), Pha (red color)); (**A**) control, (**B**) free Pha, (**C**) CH_3_-PEAH-UCNPs-Pha, and (**D**) FA-PEAH-UCNPs-Pha, scale bars represent 20 μm; (**E**) average fluorescence intensities of Pha in the confocal images; (**F**) flow cytometry assay of free Pha, CH_3_-PEAH-UCNPs-Pha and FA-PEAH-UCNPs-Pha in MCF7 cells.

The cellular uptake and accumulation in malignant tissues of PS greatly affect the efficiency of PDT treatment [46]. The fate of the UCNP-based carriers in tumor cells was observed using a confocal laser scanning microscopy. Figure 8 exhibited the confocal microscopy images based on the blue fluorescence from DAPI bound to the nucleus, F-actin in the cytoplasm stained by Alexa Fluor 488 phalloidin, and the red fluorescence of Pha.

For MCF7 cells treated with free Pha, the red fluorescence signal from Pha detected in MCF7 cells was relatively weak (Figure 8B). On the other hand, UCNP-based Pha carriers, FA-PEAH-UCNPs-Pha and CH_3_-PEAH-UCNPs-Pha, showed higher red fluorescence Pha signal compared with the free Pha sample (Figure 8C,D). Especially, after FA-PEAH-UCNPs-Pha treatment, MCF7 cells exhibited strong red fluorescence of Pha around the nucleus and inside the cells. It means that Pha was effectively internalized into the MCF7 cells (Figure 8D). To compare the cellular uptake difference between CH_3_-PEAH-UCNP-Pha and FA-PEAH-UCNP-Pha, the fluorescence intensities of Pha in the confocal images was analyzed by dividing the local image into six equal areas and the average fluorescence intensities were plotted. Free Pha and CH_3_-PEAH-UCNP-Pha samples showed similar fluorescence intensities of Pha, while FA-PEAH-UCNP-Pha showed much stronger fluorescence intensity (Figure 8E). As shown in Figure 8F, flow cytometry assay also showed that the cellular uptake of FA-PEAH-UCNPs-Pha higher than that of Free Pha and CH_3_-PEAH-UCNPs-Pha, in agreement with the confocal microscopy results.

The results indicate that the UCNP-based Pha carrier improved the water solubility of Pha molecules, and FA ligands could enhance the cellular uptake of Pha into MCF7 cells by an active targeting effect. In addition, these strong fluorescence signals from Pha molecules can be used to determine the dynamics of signal transduction in the intracellular networks and in diagnostics.

### 3.6. In Vitro Phototoxicity of UCNP-Based Nanocarriers

**Figure 9:**
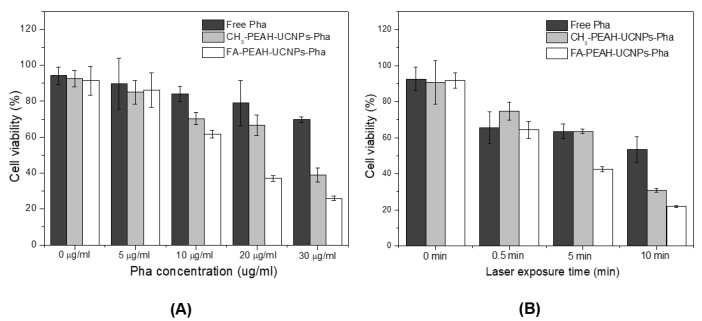
In vitro cytotoxicity test using free Pha and FA/CH_3_ -PEAH-UCNPs-Pha against MCF7 cells, (**A**) phototoxicity depending on the Pha concentration; (**B**) phototoxicity depending on laser exposure time.

To evaluate the PDT efficacy of the UCNP-based Pha nanocarrier, the in vitro cytotoxicity of FA-PEAH-UCNPs-Pha, CH_3_-PEAH-UCNPs-Pha, and free Pha was measured. For the phototoxicity test, we investigated the phototoxicity against MCF7 cells using various concentrations of Pha (0, 5, 10, 20, and 30 μg/mL) and laser exposure times (0, 0.5, 1, and 5 min). After 980 nm laser radiation for 5 min at 0.1 mW/cm^2^, FA-PEAH-UCNPs-Pha and CH_3_-PEAH-UCNPs-Pha exhibited significantly enhanced phototoxicity compared to free Pha (Figure 9A).

As the Pha concentration increased, the cell viability gradually decreased. Notably, the viability of MCF7 cells treated with FA-PEAH-UCNPs-Pha sample decreased to nearly 25% after a 5 min treatment at a concentration of 30 μg/mL. In order to determine the effect of laser exposure time on the phototoxicity, we also determined the in vitro phototoxicity of free Pha, FA-PEAH-UCNPs-Pha, and CH_3_-PEAH-UCNPs-Pha after 980 nm laser radiation for 0, 0.5, 1 and 5 min at 0.2 mW/cm^2^ (10 μg/mL, Pha equiv.). Under the dark condition, free Pha, FA-PEAH-UCNPs-Pha, and CH_3_-PEAH-UCNPs-Pha exhibited more than 90% cell viability and no significant dark toxicity as shown in Figure 8B (0 min of laser exposure time, 10 μg/mL Pha concentration). However, the cell viability significantly decreased as the laser exposure time increased (Figure 9B). The PDT efficiency of FA-PEAH-UCNPs-Pha was obviously higher than free Pha and CH_3_-PEAH-UCNPs-Pha. These results are probably due to the increased solubility of hydrophobic Pha molecules in aqueous environments by loading into the block copolymer chain-immobilized UCNP carriers, resulting in an enhanced ^1^O_2_ quantum yield of Pha.

Several papers have reported that targeting using folic acid receptors does not seem to have a significant effect on increasing phototoxicity [47]. However, many studies have reported that active targeting using folic acid ligands increases phototoxicity [48,49,50]. In this study, FA-conjugated FA-PEAH-UCNPs-Pha showed higher phototoxicity than free Pha and CH_3_-PEAH-UCNPs-Pha, possibly due to improved cellular uptake by the FA receptor-medicated endocytosis process.

## 4. Conclusions

In order to demonstrate the NIR-light induced photodynamic therapy, we have designed the NIR light-triggered theranostic system based on hexagonal-phase UCNPs for efficient PDT with enhanced deep tissue penetration ability and fluorescence imaging. Hexagonal-phase NaYF4:Yb/Er UCNPs were prepared by a hydrothermal method and the UCNPs were monodisperse with a uniform size of about 20 nm. The crystalline morphology of the synthesized NaYF_4_:Yb/Er UCNPs showed a thermodynamically stable hexagonal β-phase. To increase the aqueous solubility and conjugate functional moieties for subsequent biological functionalization, the surface of UCNPs was modified with FA-conjugated copolymers through a bidentate dihydrolipoic acid linker. The FA-PEAH copolymer-modified UCNPs (FA-PEAH-UCNPs) exhibited enhanced solubility in aqueous solution. Then, the hydrophobic Pha molecules were combined to FA-PEAH-UCNPs. These water dispersible FA-PEAH-UCNPs have a much stronger luminescence property compared with hydrophobic UCNPs. The upconversion fluorescence spectra of FA-PEAH-UCNPs excited with a 980 nm laser showed sharp green emissions at 510~530 nm and at 530~570 nm as well as a red emission at 645~680 nm. These FA-PEAH-UCNPs-Pha that produce high energy visible photons from low energy radiation in the NIR region could be promising materials for PDT and bioimaging.

Due to the active tumor targeting FA ligand conjugation, the phototoxicity and cellular uptake against MCF7 cells of FA-PEAH-UCNPs-Pha were significantly enhanced compared with free Pha and FA-ligand unconjugated CH_3_-PEAH-UCNPs-Pha. These UCNP-based Pha nanocarriers, which include FA-conjugated copolymers, could increase the aqueous solubility of hydrophobic Pha molecules and enhance ^1^O_2_ quantum yield of tumor tissue, as well as improve PDT efficiency. In addition, this UCNP nanocarrier could be triggered by NIR which has low photodamage effects on cells, and high signal-to-noise ratios, and deep tissue penetration. These results indicate that the FA-PEAH-UCNPs-Pha system could have great potential to serve as a targeted PS delivery system for tumor PDT applications in deep tissue.
